# Human Infection with *Pseudoterranova azarasi* Roundworm

**DOI:** 10.3201/eid1703.101350

**Published:** 2011-03

**Authors:** Naoki Arizono, Toshiyuki Miura, Minoru Yamada, Tatsuya Tegoshi, Kotaro Onishi

**Affiliations:** Author affiliations: Kyoto Prefectural University of Medicine, Kyoto, Japan (N. Arizono, M. Yamada, T. Tegoshi, K Onishi);; The University of Tokyo, Tokyo, Japan (T. Miura)

**Keywords:** Pseudoterranova, Anisakis, anisakiasis, pseudoterranovosis, nematode infections, identification, parasites, gastrointestinal tract, letter

**To the Editor:** Eating raw or undercooked marine fish may lead to infection with several helminths. Members of the *Pseudoterranova decipiens* species complex are the second most common nematodes found in humans (most common are nematodes of the *Anisakis simplex* complex) (*1*,*2*). The *P. decipiens* species complex consists of at least 5 sibling species (genetically but not morphologically distinguishable): *P. decipiens* sensu stricto, *P. azarasi, P. cattani, P. krabbei*, and *P. bulbosa*. In northern Japan, human infection with *Pseudoterranova* spp. is not rare; by the mid-1990s, as many as 769 cases had been reported (*3*). Pseudoterranovosis has also been encountered in North and South America and Europe (*4*,*5*). However, possible biologic and geographic differences of the sibling species of genus *Pseudoterranova* in relation to human infection remain unknown. We report a case of pseudoterranovosis for which the sibling species was confirmed as *P. azarasi*.

In 2009, a woman in Japan coughed up a nematode and expelled it through her mouth. Her medical history was unremarkable, and she had not traveled abroad for the past few years. Measurements of the worm were as follows: body 35 mm long and 0.85 mm wide; esophagus 1.88 mm long; ventriculus 1.05 mm long; and intestinal cecum 0.95 mm long, extending anteriorly along the ventriculus. The anterior end of the worm contained 3 lips. The tail was conical, 0.21 mm long, and had a small, knob-like process at the posterior end. On the basis of morpohologic features, the worm was identified as a 4th-stage larva of *P. decipiens* (sensu lato) roundworms.

DNA was extracted from the isolate from the patient (clinical isolate) and from isolates of *P. decipiens* (sensu lato) larvae from Pacific cod purchased at a local market. PCR was performed and the amplification products were directly sequenced. Primers used were 5′-CCGGGCAAAAGTCGTAACAA-3′ and 5′-ATATGCTTAAATTCAGCGGGT-3′ for a region that spans the internal transcribed spacer (ITS) 1, ITS2, and 5.8S rRNA; 5′-CTACTACTAAGAATTTGCGT-3′ and 5′-AATCCAAATACTTACGAGGA-3′ for cytochrome oxidase subunit 1; and 5′-CAGCGTATTGGTCCTAATAA-3′ and 5′-AGCATAAACAAAAGTAAACTCA-3′ for NADH dehydrogenase subunit 1.

Nucleotide sequences for the clinical and Pacific cod isolates (GenBank accession nos. AB576756–AB576761) were compared with those in DNA databases available to the public. The ITS1 and ITS2 sequences of the clinical and Pacific cod isolates were identical to those of *P. azarasi* roundworms; however, the ITS sequences of these 2 isolates also showed close similarity to those of *P. decipiens* (sensu stricto) worms and differed by only 1 nt in ITS2. However, phylogenetic tree analyses of NADH dehydrogenase subunit 1 and cytochrome oxidase subunit 1 sequences showed that the clinical and Pacific cod roundworm isolates clustered with *P. azarasi* and were clearly distinguished from *P. decipiens* (sensu stricto), showing that the clinical isolate belonged to *P. azarasi* (Figure).

**Figure:**
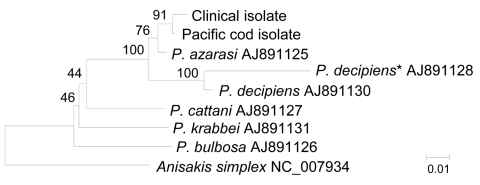
Phylogenetic analysis of members of *Pseudoterranova decipiens* species complex roundworms. Genetic relationships between NADH dehydrogenase subunit 1 sequences in clinical and Pacific cod isolates and species were inferred by using the neighbor-joining method. Bootstrap values (1,000 replicates) are shown next to the branches. The final dataset contained 498 positions. *P. decipiens sensu stricto*.* Scale bar indicates nucleotide substitutions per site.

Geographic distribution of the 5 sibling species of the *P. decipiens* complex differs somewhat. *P. azarasi* and *P. bulbosa* are found in northwestern Pacific (including Japan), *P. decipiens* (sensu stricto) and *P. krabbei* in northeastern Atlantic, *P. decipiens* (sensu stricto) in northwestern Atlantic, and *P. cattani* in southeastern Pacific waters (*1*). Given this distribution, it is not surprising that the clinical isolate and the larvae from the Pacific cod were identified as *P. azarasi*, 1 of 2 species found in water near Japan. Adult worms live in the intestines of seals and sea lions, and infective larvae live in the tissues of various marine fish, including cod, pollack, and smelt (*1*).

In Japan, most patients infected with *Pseudoterranova* spp. have acute or subacute abdominal pain, and larvae are extracted from the stomach endoscopically. However, for some patients, diagnosis is made when 4th-stage larvae are expelled from the mouth, indicating that the larvae developed from the 3rd to 4th stage during the time of infection, as did the worm reported here. Expulsion of *Pseudoterranova* spp. larvae from the mouth in the absence of severe gastric symptoms occurs more commonly in Chile (*5*). Whether the varied symptoms triggered by infection with *Pseudoterranova* spp. larvae reflect different responses of individual hosts to the worms or whether the pathogenicity of *Pseudoterranova* spp. in humans differs among worm species remains to be elucidated.

Because of the increasing worldwide popularity of eating sushi and sashimi made of raw marine fish, consumers should be made aware of the possible risk for fish-borne parasitoses. Freezing and storing fish at −20°C for 7 days or freezing at −35°C until solid and storing at −35°C for 15 hours is sufficient to kill parasites (*6*).
